# Evaluation of immune sensor responses to a viral small noncoding RNA

**DOI:** 10.3389/fcimb.2024.1459256

**Published:** 2024-10-08

**Authors:** Mehmet Kara, Scott A. Tibbetts

**Affiliations:** ^1^ Department of Molecular Biology and Genetics, Faculty of Arts and Sciences, Bursa Uludag University, Bursa, Türkiye; ^2^ Department of Molecular Genetics and Microbiology, College of Medicine, University of Florida, Gainesville, FL, United States

**Keywords:** murine gammaherpesvirus 68, Toll-like receptor, TLR4, noncoding RNA, HEK-blue TLR reporter cells

## Abstract

**Introduction:**

Gammaherpesviruses are widespread pathogens causing persistent infections linked to the development of numerous types of lymphomas in humans. During latency, most of the viral protein-coding genes are suppressed, facilitating evasion of adaptive immune recognition of protein antigens. In contrast, many noncoding RNA (ncRNA) molecules are expressed in infected cells and can regulate key cellular pathways while simultaneously evading adaptive immune recognition. To counteract this, many cells express internal pattern recognition receptors that can intrinsically sense ongoing infections and initiate cellular defenses. Murine gammaherpesvirus 68 (MHV68) is a valuable model to study *in vivo* aspects of gammaherpesvirus pathogenesis. The MHV68 ncRNA TMER4 (tRNA-miRNA-encoding RNA 4) promotes lymph node egress of infected B cells: in the absence of TMER4, MHV68-infected B cells accumulate in the lymph node in a manner similar to B cells activated through specific antigen encounter.

**Method:**

We hypothesized that TMER4 may alter intrinsic immune activation. In research described here, we aimed to explore the immunomodulatory functions of TMER4 by evaluating its impact on signaling through the critical immune sensors Toll-like receptor 4 (TLR4), TLR3, TLR7, and retinoic acid-inducible gene I (RIG-I). To accomplish this, we developed a system to test noncoding RNAs using commercially available reporter cell lines. We optimized the experimental procedure to ensure ncRNA expression and to quantify immune sensory molecule induction or inhibition by the expressed ncRNA.

**Results and discussion:**

Expression of TMER4 RNAs from plasmid constructs did not alter TLR or RIG-I signaling. This study provides a clear experimental framework that can be applied to test other small ncRNAs for their impact on various innate immune sensor proteins.

## 1 Introduction

Research on the regulation of cellular mechanisms driven by noncoding RNA (ncRNA) molecules has been gaining interest in recent years. There are different sizes of regulatory ncRNAs ranging from 20 nucleotide (nt) miRNAs to over 100 kb long ncRNAs (lncRNAs) (Bartel, 2004; Furuno et al., 2006). Many of these ncRNAs play different roles in transcriptional and posttranscriptional control of gene expression, chromatin loop formation, epigenetic modifications, and scaffolding for protein-protein, protein-RNA/DNA complex formations (Mattick et al., 2023). Remarkably, some of the first identified ncRNAs are expressed by viruses such as adenovirus-associated RNAs (VA RNAI and II) (Ohe et al., 1969), Epstein-Barr Virus (EBV) encoded small RNAs (EBERs) (Howe and Shu, 1989), and Herpesvirus Saimiri (HVS) U-rich RNA (HSURs) (Lee and Steitz, 1990; Albercht and Fleckenstein, 1992). The molecular functions of such viral ncRNAs and their relevance in virus biology are not yet well understood. Further, it is highly challenging to study the molecular mechanisms by which these viral ncRNAs function in the context of *in vivo* infections.

Gammaherpesviruses are associated with the development of lymphoproliferative diseases and several types of lymphomas in immunocompromised individuals. They infect their hosts and establish lifelong chronic infections, called latency, predominantly in lymphoid cells, mainly B cells. During latent infection, most of the viral protein-coding genes are silenced, while a vast range of ncRNAs is expressed to regulate cellular pathways related to the maintenance of chronic viral infection, blocking apoptosis, promoting tumorigenesis and modulation of immune responses (Wang et al., 2021). The ncRNAs within the cell are generally nonimmunogenic compared to viral proteins, which are presented on the cell surface by major histocompatibility complexes. Thus, the regulation of cell processes by viral ncRNAs is thought to be a part of viral immune evasion strategies (Kincaid and Sullivan, 2012). The two human gammaherpesviruses, EBV and Kaposi’s Sarcoma-associated Herpesvirus (KSHV) generate numerous ncRNA molecules, including miRNAs (Chandriani et al., 2010; O’Grady et al., 2016; Boldogkői et al., 2019). Research on the role of ncRNA function in the pathogenesis of these viruses is minimal due to the high host specificity. Murine gammaherpesvirus 68 (MHV68) infects laboratory mice and causes diseases similar to EBV and KSHV. It provides a valuable model system for understanding the molecular functions of virally encoded ncRNAs during pathogenesis (Efstathiou et al., 1990; Barton et al., 2011; Wang et al., 2021). Indeed, MHV68 encodes a unique class of ncRNAs which are 200-250 nt linked tRNA-miRNA elements called TMERs. Initially, these ncRNAs were identified as nonaminoacylated viral tRNAs (Bowden et al., 1997; Virgin et al., 1997). The TMERs are constitutively expressed in infected cells even during latency, and thus have been used as *in situ* hybridization probes to detect virus-positive latently-infected and tumor cells in mice infected with MHV68 (Simas and Efstathiou, 1998).

MHV68 encodes 8 TMER genes, all clustered in the left end of the genome. The tRNA-linked pri-miRNA sequences are transcribed by RNA polymerase III and form a predicted tRNA cloverleaf structure linked to one or two pre-miRNA stem-loops. Subsequent tRNase Z digestion separates the tRNA from the stem-loops, and each stem-loop can be processed by Dicer to generate two mature miRNAs (Bogerd et al., 2010; Diebel et al., 2010). This results in 28 mature miRNAs from 8 TMERs in the MHV68 genome (Reese et al., 2010; Bullard et al., 2019). Aside from the miRNAs and tRNAs generated from these elements, intermediate RNA structures of TMERs may function as ncRNA (Feldman et al., 2016; Hoffman et al., 2019), similar to EBV- EBERs and Adenovirus-VA RNAs because of their similar characteristics and abundance in the infected cell.

MHV68 TMER4 plays a significant role in the establishment of latency: Viruses deficient in TMER4 are severely impaired in dissemination from the initial draining lymph node, where the virus undergoes initial seeding of naïve B cells, to peripheral secondary lymphoid organs, where latency is established (Feldman et al., 2016). This critical function in B cells is carried out by a 140 nt TMER4 intermediate species that is comprised of the vtRNA plus the first stem loop, completely independent of the primary miRNA sequence (Hoffman et al., 2019). Notably, while naïve B cells normally traffic in and out of lymph nodes, B cells that are activated in the lymph node remain at this site (reviewed in Gonzalez et al., 2011). Together, these findings led us to hypothesize that the TMER4 intermediate species may function to regulate intrinsic activation of infected B cells through interaction with innate immune sensors.

Innate immune sensors are the key players in the development of an immune response to invading pathogens and specific damage signals. These sensor molecules, such as Toll-like receptors (TLR), act as detectors that can recognize unique molecular patterns on the surface of pathogens. TLR4, for instance, recognizes bacterial cell wall component lipopolysaccharide (LPS) on gram-negative bacteria and triggers a potent inflammatory response to eliminate the invading bacteria (Kawasaki and Kawai, 2014). Although TLR4 is well-known for its ability to binds and respond to the LPS, its activation through virus infections has been reported (Kurt-Jones et al., 2000; Morris et al., 2007). Moreover, in the context of herpesvirus infection TLR4 is known to induce gene expression that inhibits lytic replication and promotes latency (Doyle et al., 2002). Thus, TLR4 poses an interesting potential target for regulation by viral ncRNAs.

To determine whether the TMER4 intermediate species altered TLR4 signaling, we tested whether TMER4 intermediate or control viral ncRNA species interacted with TLR4 or other innate immune sensors such as TLR3, TLR7 and RIG-I. Experimental conditions for testing this viral RNA were extensively optimized and validated. Though TMER4 RNA was expressed in high amounts at early time points, this ncRNA neither activated nor inhibited mouse TLR3, TLR4, TLR7 or RIG-I. Additionally, we report this ncRNA was present in nuclear RNA fraction at high levels in latently infected B cells, suggesting its potential role in the regulation of gene expression.

## 2 Materials and methods

### 2.1 Cell culture and cell lines

The HEK-Blue mouse TLR (catalog no: hkb-mtlr3, hkb-mtlr4, hkb-mtlr7) and HEK-Lucia (hkl-hrigi) cell lines present in this study were obtained from InvivoGen (San Diego, CA). The mouse B cell line A20 was obtained from American Type Culture Collection (ATCC) and the latently infected HE2.1 cell line was obtained from J. Craig Forrest Laboratory (Forrest and Speck, 2008).

Human embryonic kidney (HEK) 293 cells were maintained in Dulbecco’s modified Eagle’s medium (DMEM) with 10% fetal calf serum, 100 U/mL of penicillin, 100 mg/mL streptomycin, and 2 mM L-glutamine. HEK-Blue mTLR4 cells (InvivoGen, catalog no:hkb-mtlr4) were maintained similarly to HEK 293 cells with the addition of HEK-Blue selection antibiotics (provided with cells at 1000x concentration) for TLR3, 4 and 7 cells. The full description for InvivoGen cell line used here can be reached at (hek_blue_mtlr4_tds.pdf, n.d.). To briefly describe the company cell line, HEK 293 cells were transduced with corresponding mouse TLR3, 4 or 7 receptor/coreceptors and Secreted Embryonic Alkaline Phosphatase (SEAP) reporter construct to generate HEK-Blue mTLR cells. Activation of TLR results in the expression of SEAP, which contains an interferon-inducible promotor. The secreted enzyme causes regular red media color to change into purple/blue in HEK Blue detection media. The color change is evaluated by 600-620 nm absorbance in a spectrometer or a plate reader. For the HEK RIG-I Lucia cells, activation of RIG-I results in expression of renilla luciferase and the signal was later detected by Quanti-Luc (InvivoGen rep-qlc4r1) reagent.

The mouse B cell lines, A20, and latently infected HE2.1 (described in [Forrest and Speck, 2008)] cells were maintained in complete RPMI 1640 with 10% fetal calf serum, 100 U/mL penicillin, 100 mg/mL streptomycin, 2 mM L-glutamine, and 50 μM beta-mercaptoethanol. HE2.1 cells were maintained under 300 μg/mL hygromycin. Reactivation of the virus was induced by 20 ng/mL 12-O-Tetradecanoylphorbol-13-acetate (TPA), a common herpesvirus reactivation reagent. The virus infection RNA control samples were prepared from the mouse fibroblast cell line NIH 3T12 which was maintained in DMEM media similar to HEK 293. Infections were done at 5 viruses per cell and 18 hours post-infection, RNA was isolated with Trizol.

### 2.2 Plasmids

Plasmids were generated on the pUC19 background. For different TMER4 versions, viral BAC DNA preparations from different viruses described by Hoffman et al. (Hoffman et al., 2019) were used as template DNA. Briefly, WT MHV68 for wild type TMER4, MHV68.CCA virus for TMER4 intermediate, MHV68.CCA.SLSS virus for TMER4 intermediate and MHV68.Δ5.6 miRNA deletion mutant for vtRNA4 plasmids. PCR amplicons were generated with T4.pUC19.Hind.FWD and T4.pUC19.Xba.REV primers (**Table 1**) and gel purified products were inserted into HindIII and XbaI digested linear pUC19 plasmid with NEBuilder HiFi DNA Assembly kit (NEB) according to manufacturer’s instructions. Reactions were then transformed into competent Top10 *E. coli*. The transformants were Sanger sequenced to validate insertion. Plasmids were amplified and plasmid DNA was isolated by NucleoSpin (Macherey-Nagel) or Endofree Maxi Kit (Qiagen) according to the manufacturer’s instructions. Similar cloning primers and procedures were conducted to obtain EBER, EBER2 and VA RNA I plasmids and their mutated versions from the viruses described by Hoffman et al (Hoffman et al., 2019). Sequences of TMER4 species and EBERs and VA RNA I were provided in the **Supplementary Table S1**.

**Table 1:** Primers used in this study.

No	Primer	Sequence 5’ to 3’
1	T4.pUC19.Hind.FWD	***ATGACCATGATTACGCC*** AAAGCTCTAAAGCTCTGGTCTG
2	T4.pUC19.Xba.REV	***CCGGGGATCCTCTAG*** AGACTTGGGACATCTGGGGG
3	TMER4.FWD	GTCGGGGTAGCTCAATTGGT
4	TMER4.T7.REV	*TAATACGACTCACTATAGGGAGA*CTGGGAAAAGAAAAAACCACCT
5	U6.Endlabel	GCTAATCTTCTCTGTATCGTTCCAATTTTAGTATATGTGCTGCC
6	5.8S.Endlabel	ACGCACGAGCCGAGTGATCCACC

In 1 and 2, bold italic sequences are homologous sequences to pUC19 HindIII and XbaI upstream and downstream digestions sites, respectively. In primer 4, the underlined italic sequence is the T7 promoter sequence.

### 2.3 Transfections, HEK-Blue and HEK lucia detection

Transfections were done in either 96-well or 12-well plates. Initially, for 96-well plate transfections, 10^4^ HEK-Blue mTLR (3, 4 or 7) cells were plated with 200 µL HEK-Blue Detection media per well on the morning of transfection day. In the evening, 100 ng of the sample plasmid was transfected with Lipofectamine 3000 (Thermo Fisher Scientific) according to the manufacturer’s instructions. Briefly for 10 wells of a 96-well plate, 1 µg of plasmid DNA and 1µL of P3000 reagent were mixed in 100 µL of OptiMEM media in an Eppendorf tube. In another Eppendorf tube, 2 µL of Lipofectamine was diluted into a total of 100 µL OptiMEM. Tubes were incubated at room temperature for 5 minutes and two tubes were mixed. The mixture was incubated for 15 minutes at room temperature. 20 µL of the reaction was added to each well. For 12-well plate transfections, 10^5^ HEK-Blue mTLR4 cells were plated with DMEM media in the morning and ~8 hours later, transfected with 1µg of plasmid with Lipofectamine 3000. The next morning cells were checked for GFP expression. DMEM media were removed and cells were gently washed with PBS once and resuspended in 1mL of HEK-Blue Detection media. 200 µL HEK-Blue Detection media-containing cell suspension was plated into a 96-well plate. 16-24 hours post-transfection (hpt), absorbance at 620 nm was measured with Promega Glomax Multi-detection System. Data were analyzed by either Excel or GraphPad Prism 6 software. For statistical analysis One-way ANOVA was used by comparing each group to empty vector control group. P values are given in **Supplementary Material** file.

For induction and inhibition assays of TLR4, 5 mg of lipopolysaccharide (LPS, InvivoGen catalog no: tlrl-eblps) was dissolved in 1 mL of ddH_2_O to a final stock concentration of 5 µg/µL. 1mg of CLI095 (tlrl-cli95) was dissolved in 1 mL of DMSO to a final stock concentration of 1 µg/µL. 100mg Polymyxin B (tlrl-pmb) was dissolved in 2 mL of ddH_2_O to a final stock concentration of 50 µg/µL. For TLR3 polyIC (InvivoGen tlrl-pic) was used at 1µg/mL for the induction and 100ng/mL for the inhibition assays. For TLR7, its ligand CLI307 was used at 1µg/mL for the induction and 100ng/mL for the inhibition assays.

HEK Lucia cells were prepared in a similar fashion to TLR cell lines and transfected. 24-48 hours post transfection, most of the media is removed leaving only 50 µL of original media. 50 µL of Quanti-Luc (InvivoGen rep-qlc4r1) detection reagent is added to each well and luciferase activity is measured immediately with Promega Glomax Multi-detection System. For the positive control 3p-hpRNA (InvivoGen tlrl-hprna) transfected (1µg/mL) into the cells.

### 2.4 Northern blots

Total RNA was isolated from cells with Trizol according to the manufacturer’s instructions. 10 μg of total RNA was loaded onto a urea-denaturing 10% polyacrylamide gel in parallel with an RNA century marker (Ambion). The gel was run at 350V for one hour in 0.5X TBE Buffer with a BioRad Miniprotean Electrophoresis system. Then, RNA was transferred to a Hybond XL nylon membrane (Life Technologies) using a BioRad Transblot apparatus at 30V for 45 minutes, then 35V for 15 minutes, and 40V for 10 minutes. The membrane was washed, RNA was crosslinked to the membrane by UV, and the membrane was stained with 0.02% methylene blue staining for visualization of RNA integrity and markers. The cross-linked membrane was then prehybridized in a rotating hybridization oven for an hour at 60°C, with ULTRAhyb (Ambion) buffer, then hybridized with the probe overnight at 60°C. The next day, the membrane was washed 3 times with 1X SSC buffer and exposed to film at 80°C for an appropriate time.

The radioactively labeled TMER4 probe was prepared with a Maxiscript T7/Sp6 kit (Thermofisher). Briefly, the probe template was PCR amplified with primers TMER4.FWD and TMER4.T7.REV (**Table 1**). T7 polymerase generates the complementary strand of the template. The template was *in vitro* transcribed with the kit (T7 enzyme) by adding 10 μCi alpha-CTP (Perkin Elmer) to the reaction for four hours at 37°C. The DNA template was digested with DNase for 20 minutes, then the reaction was stopped by adding EDTA. The probe was used without purification. For U6 RNA and 5.8S rRNA detection end-labeling protocol was used. U6.Endlabel or 5.8S.Endlabel antisense oligo sequences were given in **Table 1**. Briefly, 1 µL of the 10 µM of the corresponding primer was labeled with gamma-ATP (Perkin Elmer) using T4 PNK at 37°C for one hour. End-labeled probes were hybridized at 40°C.

### 2.5 Nuclear cytoplasmic RNA fractionation

A total of 2x10^6^ A20, HE2.1, or TPA-reactivated HE2.1 cells were washed and centrifuged at 500xg for 5 minutes at 4°C. Cells were resuspended in 400 μL of Buffer 1(0.32 M Sucrose, 3 mM CaCl2, 2 mM MgCl2, 0.1mM EDTA, 10mM Tris pH8, 0.5% Igepal, 1 mM DTT 0.4U/μL RNasin). Cells were then incubated on ice for 5 minutes and centrifuged at 500xg for 5 minutes at 4°C. The supernatant was transferred to a new Eppendorf tube and an equal volume of Trizol was added. The pellet was gently washed with Buffer 1 once more and centrifuged without incubation. The pellet was resuspended in 400 μL of Buffer 2 (150 mM NaCl, 50 mM, 5 mM, 0.1% Triton, 0.1% SDS) and an equal volume of Trizol was added to the samples for RNA isolation.

## 3 Results

### 3.1 Cellular localization of TMER4 RNA forms

The location of a small RNA within a cell, whether cytoplasmic or nuclear, plays a critical role in its function. Cytoplasmic small RNAs, like microRNAs (miRNAs), are often involved in post-transcriptional regulation to inhibit protein translation. In contrast, nuclear small RNAs might be involved in earlier stages of gene expression, such as regulating the processing or splicing of pre-mRNA molecules before they are exported to the cytoplasm for translation. This compartmentalization ensures that these small RNAs are present in the correct location to interact with their intended targets and regulate gene expression efficiently. Understanding the specific location of a small RNA within the cell is therefore essential for deciphering its function and its role in various cellular processes.

**Figure 1:**
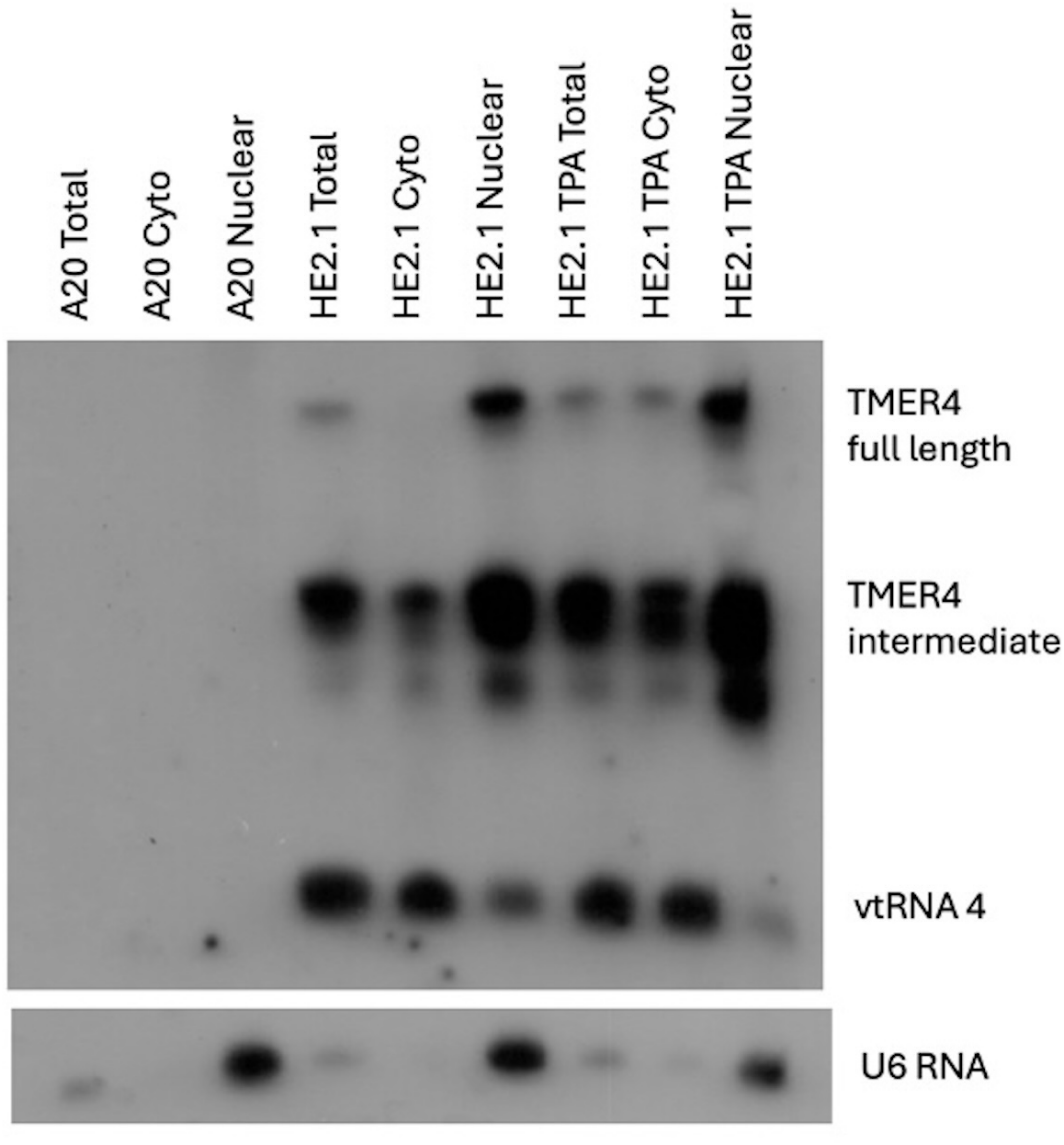
Cellular localization of TMER4 RNA intermediates by northern blot. A20 cells were the background uninfected cell line. HE2.1 cells were latently infected mouse B cell lines, carrying viral genome, generated on the A20 cell background. The full length TMER4 and the intermediate forms were enriched in nuclear RNA fraction in latently infected B cells. vtRNA is present in the cytoplasmic fraction. TPA was added at 20 ng/mL concentration to induce reactivation of the virus. U6 RNA is an exclusively nuclear RNA and was used as control for the cytoplasmic nuclear RNA fractionation procedure. Radiolabeled-*in vitro* transcribed full length TMER4 RNA antisense probe was used for blotting.

To understand the cellular localization of TMER4 RNA forms, we utilized latently infected murine B cell lines, HE2.1 cells (Forrest and Speck, 2008). B cells are the major latency reservoir for the virus and are essential for dissemination of the virus from the primary site of infection to the blood stream (Usherwood et al., 1996). In order to characterize TMER4 function we determined the location of all 3 major forms of TMER4 using northern blot of RNA following cytoplasmic and nuclear fractionation. TMER4 vtRNA4, a 74 nt processed viral tRNA, was detectable in the cytoplasmic fraction. The 203 nt full-length TMER4, which is composed of the vtRNA plus two downstream stem-loop structures, was primarily present in the nuclear fraction. Interestingly, the 140 nt intermediate form of TMER4, which consists of the vtRNA plus first stem-loop (Hoffman et al., 2019) was found in both the cytoplasmic and nuclear fractions as compared to U6 control RNA which is found only in nuclear fraction (**Figure 1**). To determine whether virus reactivation altered localization, infected B cells were induced with TPA (12-O-tetradecanoylphorbol-13-acetate); however, no TMER4 RNA forms demonstrated significantly altered localization in reactivated cells. These findings indicate that the critical TMER4 intermediate species localizes to a cytoplasmic compartment that is distinct from that of the fully nuclear full-length TMER4. This observation is fully consistent with the ability of the intermediate form of TMER4 to carry out an essential function that cannot be reproduced by full-length TMER4 (Feldman et al., 2016; Hoffman et al., 2019). Based on these findings, we investigated the possibility that the intermediate TMER4 species may activate or block cytoplasmic immune sensors.

### 3.2 Optimization of conditions to test TMER4 RNA in TLR reporter cell lines

**Figure 2:**
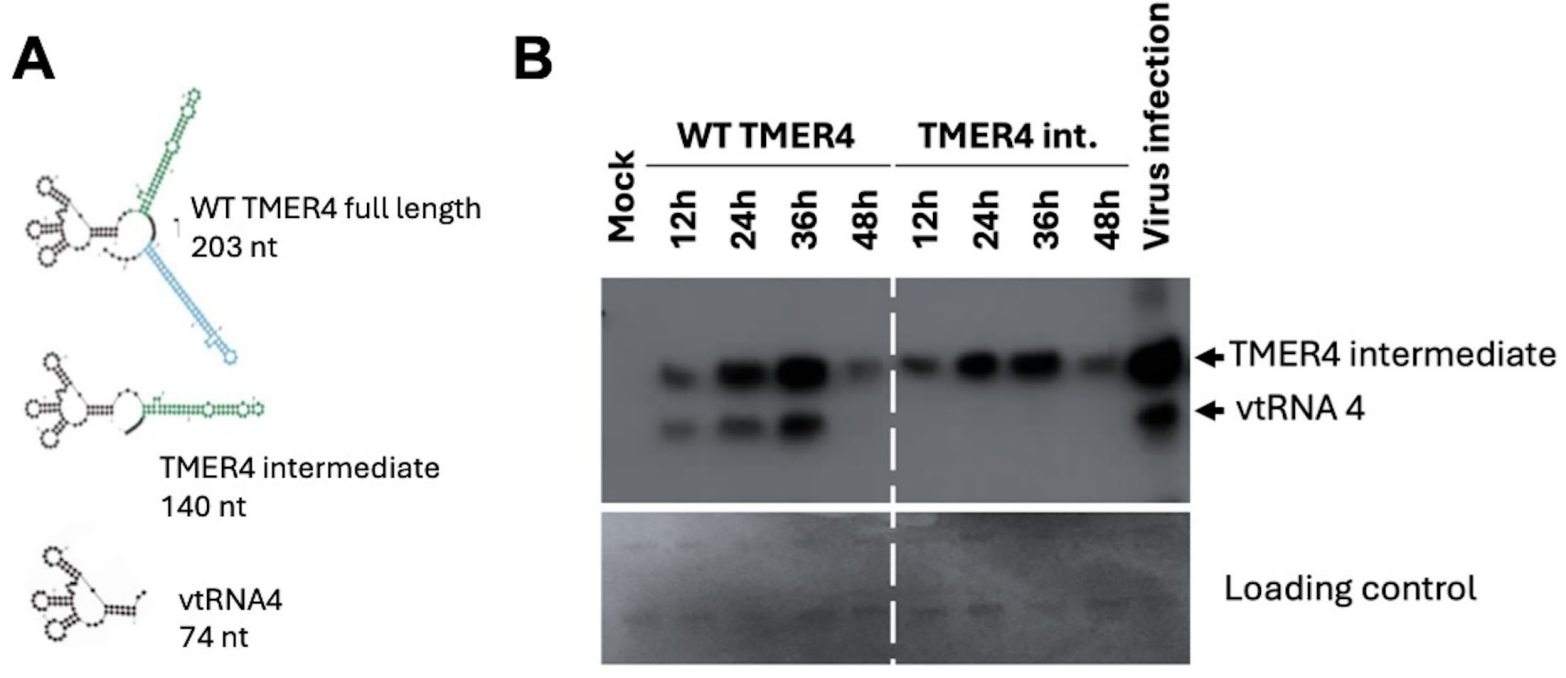
TMER4 structure and expression from pUC19 plasmid constructs. **(A)** mFold RNA structure predictions are depicted for different forms of TMER4-related RNAs. The black part is the tRNA, and green and blue stem-loops are the pre-miRNA structures. **(B)** RNA samples were prepared at the indicated time points. HEK293 cells were transfected in 12-well plates in DMEM media. 140 nt-long T4 intermediate form and 70 nt-long vtRNA were detectable at 12 hpt and peaked at 36 hpt. RNA for viral infection control was prepared from wild type virus infected fibroblasts at 18 hours post infection.

The three major forms of TMER4 RNA were analyzed *in silico* by Mfold. Structural analysis of the 203 nt full-length TMER4 sequence shows that a tRNA cloverleaf motif is linked to two miRNA-producing stem-loops (**Figure 2A**). Sequential removal of the stem-loops results in the 140 nt TMER4 intermediate form and the 74 nt vtRNA. Because TMER4 expresses these three RNA forms from the primary RNA, but only the intermediate form conveys the essential *in vivo* function (Feldman et al., 2016; Hoffman et al., 2019), to optimize functional testing we generated an artificial TMER4 sequence that expresses only the intermediate form in a plasmid backbone. This was achieved by (a) addition of CCA nucleotides into the region between the vtRNA and the first stem-loop to disable tRNaseZ processing, and (b) insertion of a strong stop sequence after the first stem-loop to prevent expression of the second stem-loop. We also generated vectors containing full-length TMER4 and vtRNA4 as controls. To validate the TMER4 species expressed by each plasmid we then transfected HEK 293 cells and performed northern blots. The TMER4 intermediate form was detectable with both full-length and TMER4 intermediate plasmids as early as 12 hours post-transfection (hpt) and remained at high levels until 36 hpt (**Figure 2B**). Transfection resulted in expression levels comparable to that of wild-type virus infection.

**Figure 3:**
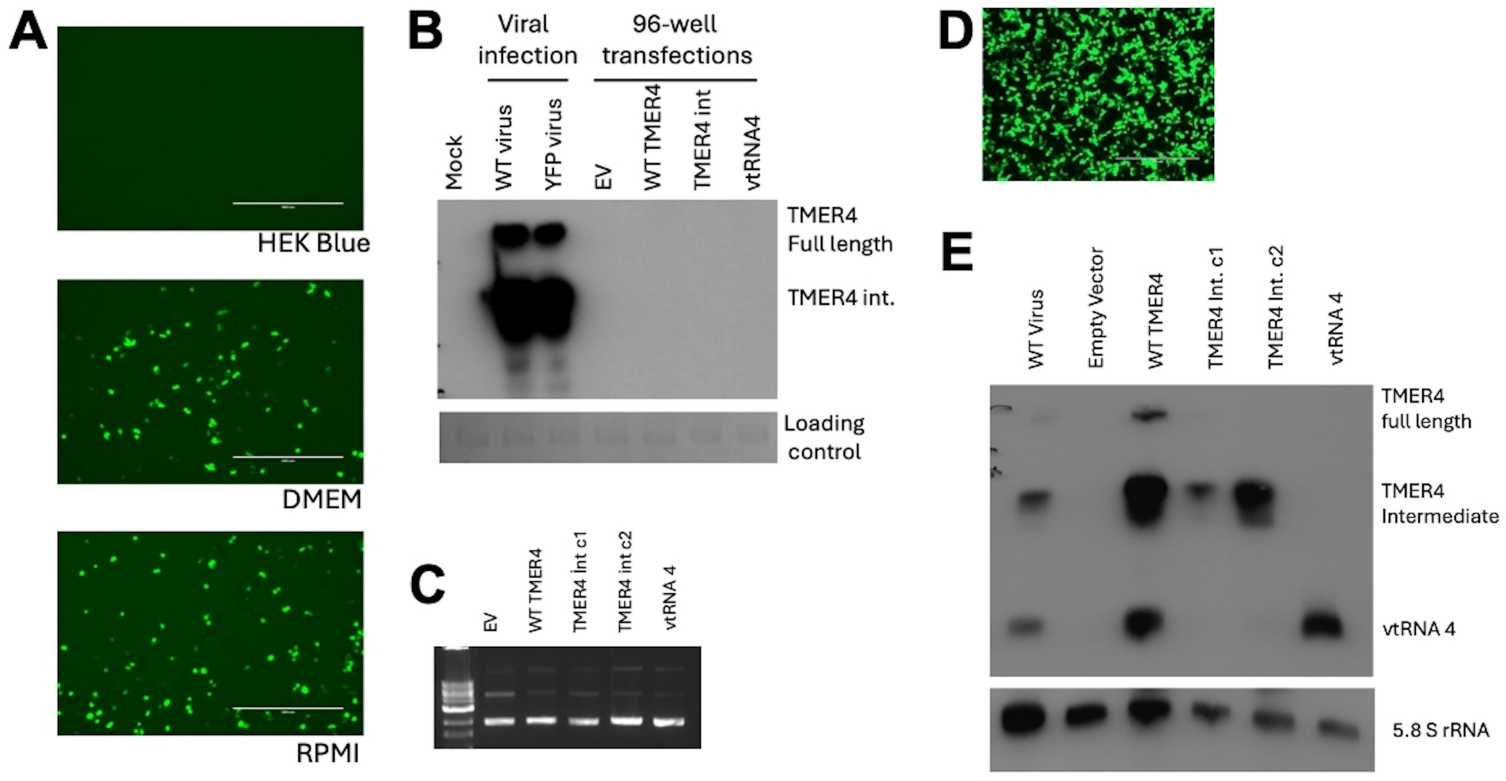
Optimization of plasmid preparations and transfections for mTLR4 reporter cells. **(A)** HEK-Blue mTLR4 cells in different media were transfected with 100 ng of pEGFP plasmid to check for transfection efficiency. Scale bar indicates 400 µm. **(B)** Northern blot analysis showed TMER4 RNA expression with indicated plasmids on top of the blot. TMER4 FL is full length TMER4 and TMER4 int is the TMER4 intermediate and two different clones of the same plasmid is used as indicated by c1 and c2. RNA from viral infections was used as a control. **(C)** Endofree (EF) plasmid kit purified plasmids contained mainly supercoiled forms in ethidium bromide containing 1% Agarose gel. **(D)** Transfection optimization with 12-well plates in DMEM media and testing for mTLR4. **(E)** Different forms of TMER4 RNA can be detected at 24 hpt and 3 different forms (full length, intermediate, vtRNA) were visible by the wild type TMER plasmid in HEK Blue mTLR reporter cells. End-labeled 5.8S rRNA was used as loading control.

To test whether TMER4 activated the cytoplasmic sensor TLR4, we attempted to transfect commercially available cell lines carrying an mTLR4 reporter with plasmids expressing TMER4 species. HEK Blue mTLR4 reporter cells stably express mouse TLR4 and activation of the receptor induces production of alkaline phosphatase reporter that can be measured in the commercially available HEK-Blue detection medium. Three different versions of TMER plasmids and the empty vector control were transfected into these cells in HEK-Blue Detection media, in accordance with the manufacturer’s protocol. However, plasmid-derived GFP signal could not be detected in the transfected cells (**Figure 3A**). Similarly, northern blot analysis confirmed that TMER4 RNA was not detectable in these samples (**Figure 3B**). In contrast, when similar transfections were performed in 96-well plates with DMEM or RPMI media instead of the HEK-Blue Detection media, GFP expression was observed (**Figure 3A**). Thus, these results indicated that transfections containing HEK-Blue Detection media were unsuitable for screening plasmid-generated RNA molecules in the TLR panel.

Because the TLR4 agonist LPS was present in DNA preparations from standard plasmid isolation kits and confounded results (**Supplementary Figure S1**), we utilized EndoFree (EF) plasmid kits to purify plasmids. The majority of plasmids were in supercoiled form, indicating high quality purification (**Figure 3C**). Thus, we confirmed that EF plasmid preparations were useable for further testing.

To further optimize transfection efficiency of TLR4 reporter cells, we performed transfections in 12-well plates in DMEM, then split the cells the following day before adding HEK-Blue detection media. At 24 hours, cells were readily transfected, as indicated by GFP expression (**Figure 3D**). To confirm RNA species expressed, we performed northern blots on RNA from transfected cells (**Figure 3E**). As expected, cells transfected with plasmids carrying WT TMER4 expressed all three TMER4 species, whereas plasmids carrying TMER4 intermediate expressed only the 140 nt intermediate species. Control plasmids carrying vtRNA4 alone expressed only the 74 nt vtRNA.

### 3.3 Testing TMER4 ncRNA species as agonists or antagonists of mTLR4

**Figure 4:**
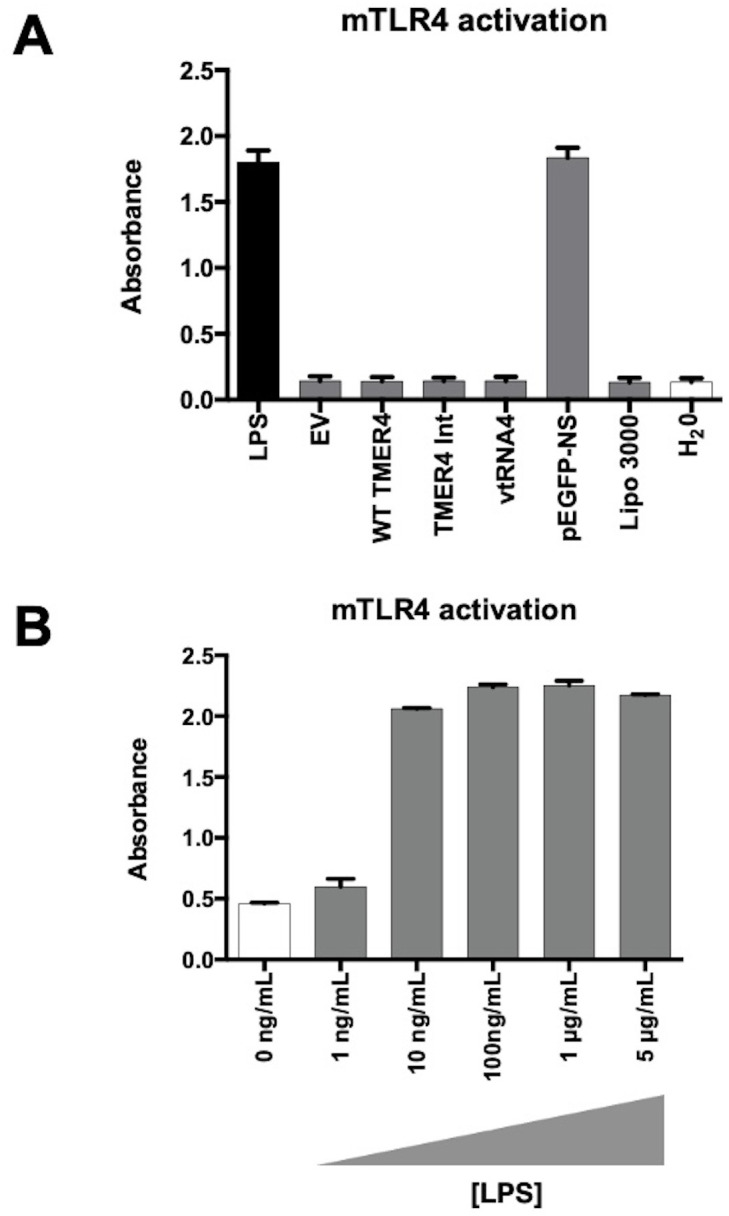
mTLR4 activation test by endotoxin-free TMER4 plasmids. **(A)** mTLR4 induction by EF plasmids was measured with HEK-Blue detection system at 600-620nm absorbance.TMER4 plasmids did not induce mTLR4 (assayed in triplicates, n=3). 100ng/mL LPS is used for induction of mTLR cells. **(B)** EV is empty pUC19 vector. pEGFP-NS plasmid is prepared with a standard miniprep kit thus contains LPS. Lipo 3000 is transfection reagent control. **(B)** Different LPS concentrations were added to cells maintained in HEK-Blue Detection Media in 96-well plates in triplicates. Statistical analysis was done by GraphPad. There was no statistical difference among tested plasmids. (p-Values > 0.99, for EV vs. pEGFP-NS p-Value **<**0.001).

To determine whether TMER4 species activated TLR4, we performed transfections of mTLR4 reporter cells with EF preparations of plasmids expressing TMER4 species, then 24 hours later measured OD 600nm absorbance (**Figure 4A**). While cells treated with the TLR4 agonist LPS induced strong activation, none of the plasmids expressing TMER4 species induced TLR4 activation as compared to empty vector (EV), lipofectamine and water controls. As a control, pEGFP plasmid prepared via standard plasmid isolation kit (NucleoSpin) showed a similar level of induction to purified LPS, confirming the presence of LPS in standard plasmid preparations. Lack of induction was not due to low sensitivity of response, as low levels of LPS induced TLR4 activation (**Figure 4B**). Together these results demonstrated that TMER4 does not activate TLR4-based signaling pathways.

**Figure 5:**
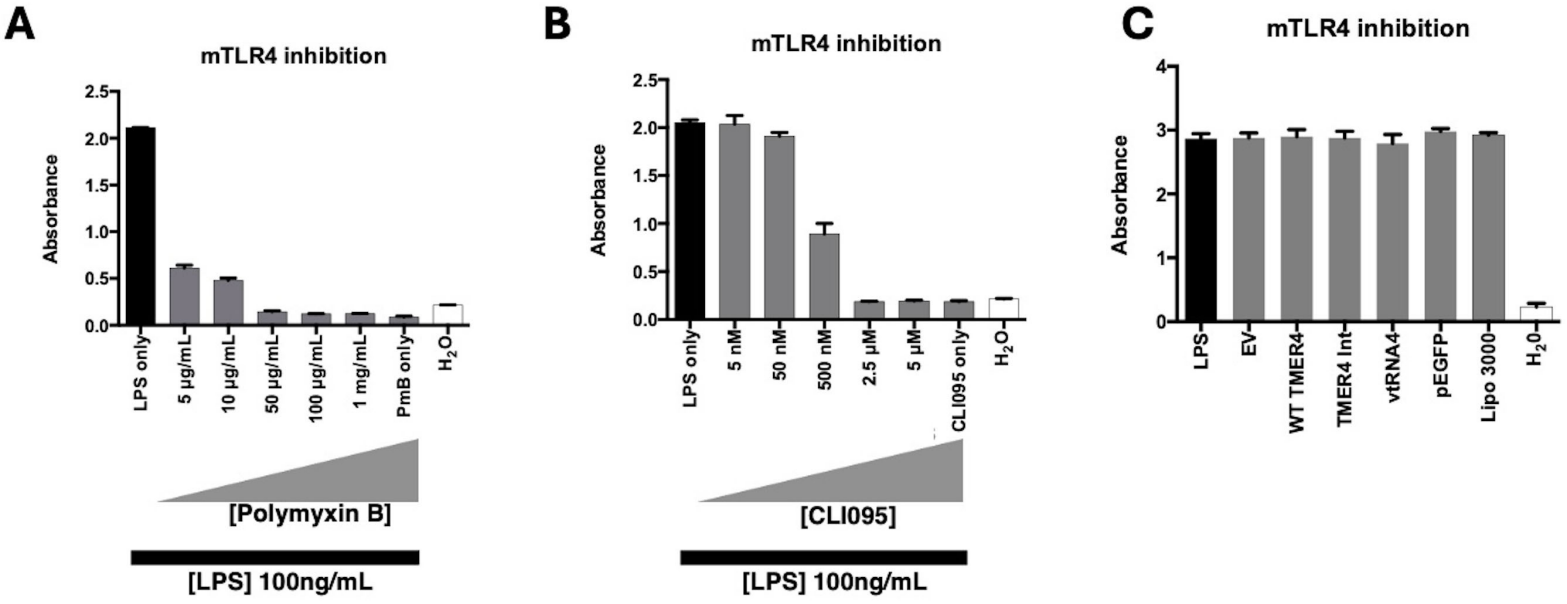
mTLR4 inhibition test by TMER4 plasmids. **(A)** LPS induction of mTLR4 was inhibited by polymxin B, an extracellular TLR4 inhibitor, at different concentrations. **(B)** LPS induction of mTLR4 was inhibited by CLI095, an intracellular TLR4 inhibitor, at different concentrations (assayed in triplicates, n=2). **(C)** None of the tested plasmids inhibited LPS-induced mTLR4 induction (assayed in triplicates, n=3). 1ng/mL LPS is used for induction of mTLR cells. Optical density (absorbance) at 600nm was shown in y axis. There was no statistical difference among tested plasmids. p-Values for empty vector vs. tested plasmids were 0.85-0.99.

To determine whether TMER4 species may instead antagonize TLR4 activation, we induced cells with LPS treatment and tested the ability of control inhibitors or TMER4 species to block activation. In control experiments, TLR4 activation was efficiently inhibited in a dose-dependent manner by both polymyxin B (PmB), an extracellular inhibitor of TLR4, and CLI095, a compound which blocks TLR intracellular signaling pathways (**Figures 5A, B**). In stark contrast, none of the TMER4 RNA species inhibited LPS-induced TLR4 activation (**Figure 5C**). Additionally, we tested EBV EBER1, 2, and Adenovirus VA RNA I plasmid constructs for TLR4 activation (**Supplementary Figure S2**). None of these RNAs induced TLR4 activation. Together, this set of experiments clearly demonstrates that TMER4 species do not act as an agonist or an antagonist for TLR4 pathways.

### 3.4 Testing TMER4 ncRNA species as agonists of TLR3, TLR7 and RIG-I

**Figure 6:**
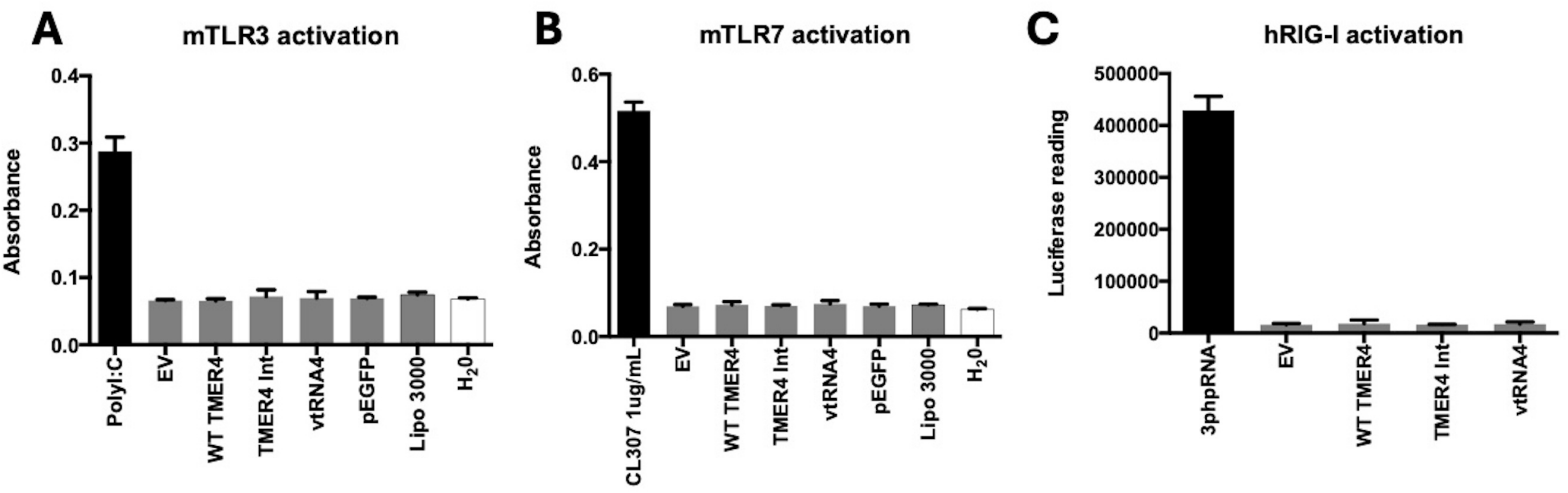
mouse TLR3, TLR7 and RIG-I activation by different TMER4 variants. **(A)** HEK-Blue mTLR3 were tested with different TMER4 variant encoding plasmids and assayed in triplicates. Absorbance was measured at 600-620nm ~16 hours post treatment (n=2). PolyI:C was used for induction at 1µg/mL **(B)** Similarly, HEK-Blue mTLR7 cell lines were assayed (in triplicates, n=2). EV is empty pUC19 vector. Lipo 3000 is transfection reagent control. CL307 was used the induction control for TLR7. **(C)** hRIG-I HEK-Lucia cell lines were assayed in triplicates (n=2). 3p-hpRNA is the positive control. Raw luciferase readings are shown in y axis. Statistical analysis was done by GraphPad. There was no statistical difference among tested plasmids. p-Values for empty vector vs. tested plasmids were ranging from 0.2 to 0.99.

In contrast to TLR4, which is a plasma membrane-associated pathogen-associated molecular pattern (PAMP) sensor, TLR3 and TLR7 are endosome-associated RNA sensor molecules. To determine whether TMER4 intermediate species induced TLR3 or TLR7, we performed similar experiments using HEK-Blue mTLR3 and mTLR7 reporter cell lines. TLR3 was strongly induced by positive control polyI:C, but was not induced by any TMER4 species (**Figure 6A**). Similarly, while TLR7 was strongly induced by agonist CL307, it was not activated by any TMER4 species (**Figure 6B**).

To determine whether TMER4 species instead blocked TLR3 activation, we attempted to perform antagonist experiments. However, if the TLR3 reporter cells were induced by polyI:C prior to plasmid transfections, the cells were not able to be transfected (not shown). In contrast, if the cells were first transfected even using control plasmids, then the polyI:C stimulation no longer induced TLR3 activation (**Supplementary Figure S3A**). Similarly, transfection of the plasmids for TLR7 inhibition did not result in a statistically significant difference with any of the TMER4 species (**Supplementary Figure S3B**).

Since TLRs are membrane-bound and associated with endosomes and plasma membrane (Kawasaki and Kawai, 2014), we also considered a role for cytosolic RNA sensor proteins. There are several cytosolic RNA sensor molecules that are activated by double-stranded RNAs produced by viruses. Melanoma differentiation-associated gene-5 (MDA5) generally binds to longer and highly structured dsRNAs. In contrast, RIG-I activation is typically associated with smaller RNAs (Radoshevich and Dussurget, 2016). To test whether TMER4 RNAs activated RIG-I, we performed agonist experiments in HEK-RIG-I Lucia cell lines. For a RIG-I agonist control, we used 3p-hpRNA, a double-stranded RNA that contains 5’ triphosphate and induces RIG-I when transfected into the cells. However, in contrast to control RNA, TMER4 RNAs did not activate the RIG-I pathway (**Figure 6C**).

Inhibition of RIG-I activation by TMER4 RNAs was also examined, but we observed no difference between the empty vector and alternative TMER4 species (**Supplementary Figure S3C**). In addition, we tested EBER1/2 and VA RNA 1 plasmid constructs for the activation of TLR3, 7 and RIG-I cell lines. We observed a dose-dependent induction of RIG-I with decreasing amounts of the synthetic 3phpRNA (**Supplementary Figure S4A**). Unfortunately, neither EBERs nor the VA RNA expressed from the plasmids could induce the HEK Blue TLR3, 7 or RIG cell lines (**Supplementary Figures S4B–D**).

## 4 Discussion

Up to now, no molecular function of MHV68-encoded TMER4 has been reported. Based on the *in vivo* biological phenotype of TMER4-deficient viruses, we speculated that this ncRNA may manipulate intracellular sensors to manipulate B cell biology. In particular, we were interested in whether TMER4 may promote or inhibit TLR4 signaling. Although TLR4 is widely known for recognizing bacterial LPS, it can also be activated by damage-associated molecular patterns and non-LPS pathogen-associated molecular patterns (Kawasaki and Kawai, 2014). The seemingly unrelated concepts of TLR4 activation and virus infection have been explored for several viruses including Ebola virus, respiratory syncytial virus and Dengue virus. In these examples, glycosylated viral cell surface proteins activated TLR4, which resulted in host gene expression beneficial for the viral infection (Kurt-Jones et al., 2000; Doyle et al., 2002; Olejnik et al., 2018). Consistent with a possibility for viral ncRNA manipulation of this pathway, multiple host small and long ncRNAs are known to promote or block TLR4 function. For example, host lncRNA *MaIL1* (macrophage interferon-regulatory lncRNA 1) is an integral component of the TLR4 signaling machinery (Aznaourova et al., 2020) while host lncRNA *MEG3* (maternally expressed gene 3) suppresses TLR4 (Tao et al., 2018). Nevertheless, despite careful analyses, we determined that the TMER4 RNAs do not activate or interfere with TLR4 signaling.

In broader interest to the field, we developed and optimized a screening method for nuclear and cytoplasmic viral ncRNAs that can be broadly used to test similar molecules for their ability to induce or inhibit innate immune signaling receptors and pathways. Cell lines such as HEK Blue reporter lines are generally used to screen small compounds for this purpose (Pérez-Regidor et al., 2022). Here we adopted this system to test viral ncRNAs by utilizing transfection of plasmids expressing viral ncRNA species in order to mimic expression from virus genomes. Although transfections in HEK Blue detection medium would be beneficial for consistency, simplicity and convenience, we found that the HEK Blue medium inhibited transfection. This problem was rectified by performing transfections in regular medium. In addition, endotoxin-free plasmid isolation kits were used to prevent LPS contamination and inappropriate activation of TLR4. The procedure described here can be used to test the effect of viral noncoding RNAs on similar innate immune sensors. For example, we also examined TMER4 modulation of TLR3 and TLR7, endosomal sensors that can be activated by dsRNA. Nevertheless, we determined that TMER4 had no effect on signaling through these membrane-associated sensory molecules.

Aside from the membrane-bound and endosome-associated TLRs (Kawasaki and Kawai, 2014), it was also important to consider whether TMER4 may alter signaling from cytosolic RNA sensor proteins. Double-stranded RNAs produced by viruses activate several cytosolic RNA sensor molecules. For example, RIG-I can be activated by highly structured, relatively short-sized dsRNA molecules (Radoshevich and Dussurget, 2016). Since the TMER4 intermediate species is detectable in the cytoplasm, it is conceivable that it plays a role in modulation of cytosolic innate immune RNA sensors, as has been reported for other viral RNAs such as the adenovirus VA RNAs and EBV EBERs. Although the specific molecular mechanisms by which these ncRNA function has not been fully elucidated, both adenovirus VA RNAs and EBV EBERs have been shown in some scenarios to bind cytosolic sensors retinoic acid-inducible gene I (RIG-I) and double-stranded (ds) RNA binding protein kinase R (PKR) to block interferon-induced apoptosis and inhibition of protein synthesis (Sharp et al., 1993; Nanbo et al., 2002; Samanta et al., 2006; Minamitani et al., 2011). Notably, expression of the EBV EBER1 or EBER2 are able to rescue the impaired B cell dissemination phenotype displayed viruses deficient in TMER4, suggesting a co-evolved function with the EBER molecules (Hoffman et al., 2019; Wang et al., 2022) despite a lack of sequence or predicted structure similarity. However, because adenovirus VA RNA does not rescue TMER4 function, it seems unlikely that TMER4 function is mediated by RIG-I binding. Consistent with this, we detected no altered RIG-I function in the presence of TMER4.

Gammaherpesvirus ncRNAs are critical regulators of the immune system during both acute infection and long-term latency. Thus, understanding the function of viral ncRNAs could help with the design of novel therapies for gammaherpesvirus diseases or biosensors for their early detection. Screening for molecular pathways altered by ncRNA expression, as we did in this study, is one potential straightforward strategy for testing function. An alternative but difficult approach is to design experiments where biochemical interactions of RNA-RNA, RNA-DNA and RNA-protein can be investigated. For example, crosslinking cells with UV or formaldehyde stabilizes RNA-associated complexes, which can be pulled down with biotinylated complementary oligonucleotides and subsequently analyzed for interacting proteins, RNA, or DNA (Chu et al., 2012; Cao et al., 2019). Future such experiments will be useful to determine how the TMER4 intermediate species promotes B cell egress and establishment of lifelong latency.

## Data Availability

The original contributions presented in the study are included in the article/**Supplementary Material**. Further inquiries can be directed to the corresponding author/s.
