# Supplementary material for: Evaluation of immune sensor responses to a viral small noncoding RNA
Source: Front Cell Infect Microbiol. 2024 Oct 8;14:1459256. doi: 10.3389/fcimb.2024.1459256 (PMC11499242; doi:10.3389/fcimb.2024.1459256)
Supplement: Supplementary file 1 [file Table1.docx]

Supplementary Material

# Supplementary Data

The original uncut images of northern blot figures is shown below for each figure.

| Figure 1. cellular localization of TMER4. Cytoplasmic vs nuclear RNA Only the red area was used for the figure. | 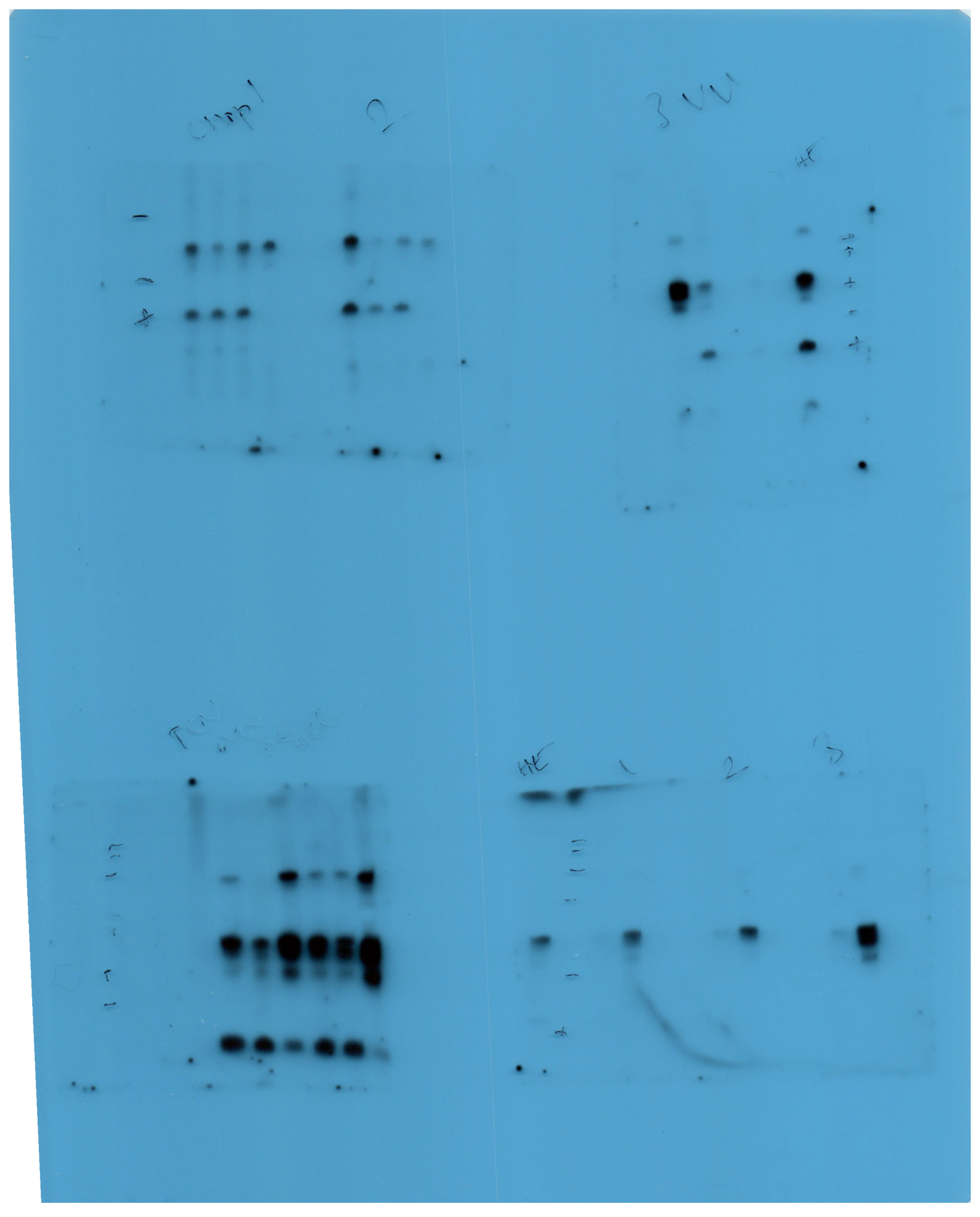 |
| --- | --- |
| Figure 1. cellular localization. Only the red area was used for the figure. Blotted for U6 RNA. | 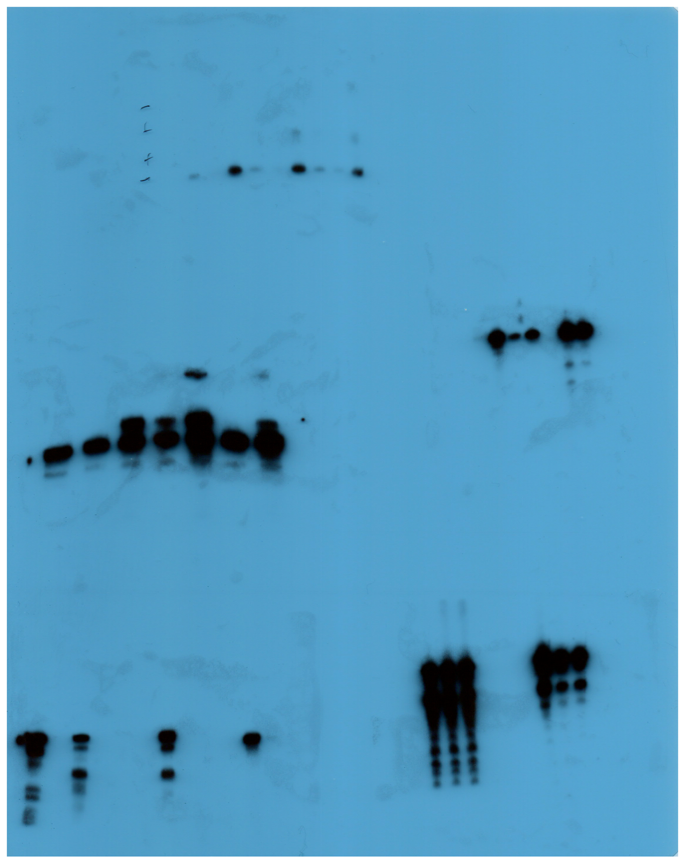 |
| Figure 2B. TMER1 expression from the TMER at different time points Only the red area was used for the figure. Blotted for TMER4 probe. | 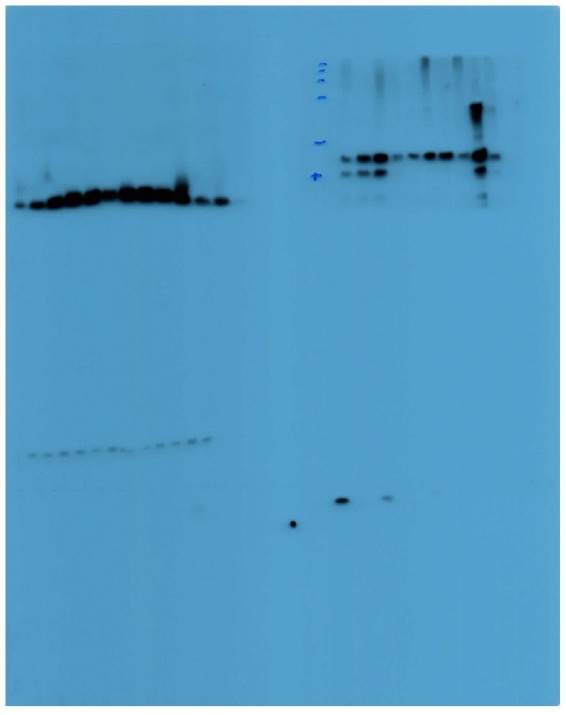 |
| Figure 3B. TMER4 expression from the plasmids in HEK Blue Detection media transfections. Only the red area was used for the figure.. Blotted for TMER4 probe. The last two lanes were plasmid controls which were not used in the figure. | 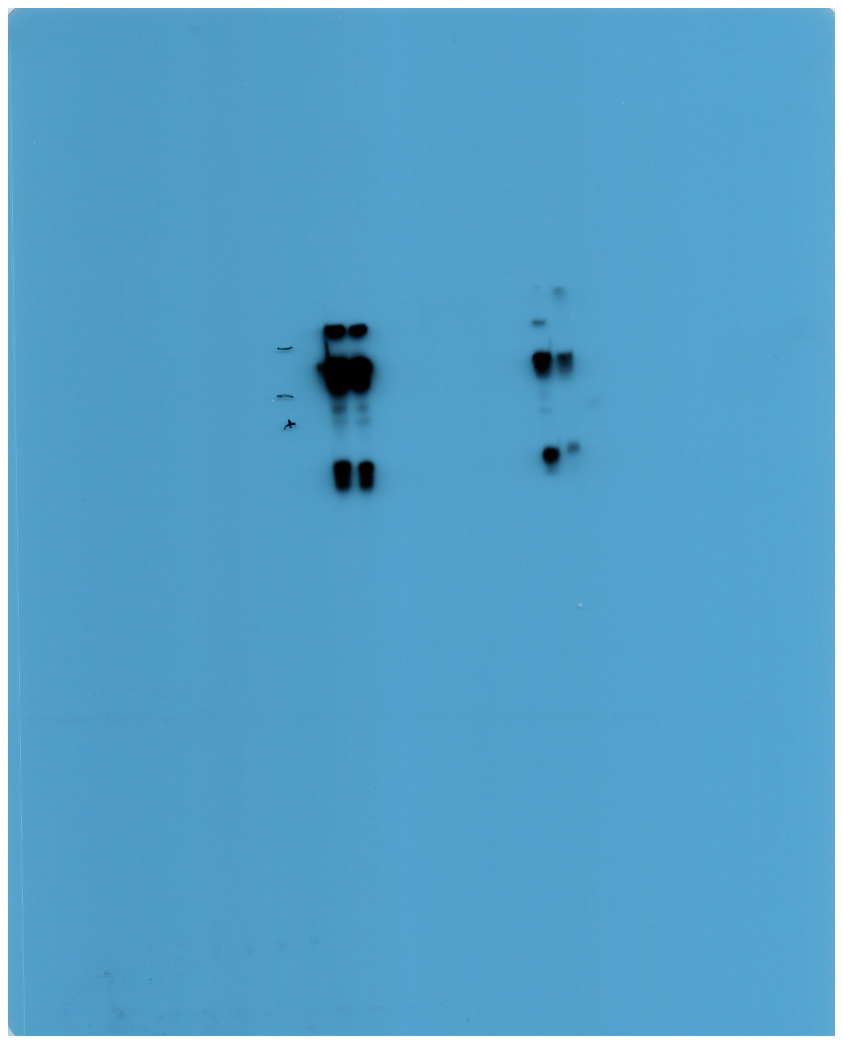 |
| Figure 3B. Loading control. The membrane was stained with Methylene Blue. Rotated version of the image was used. Blotted for TMER4 probe. | 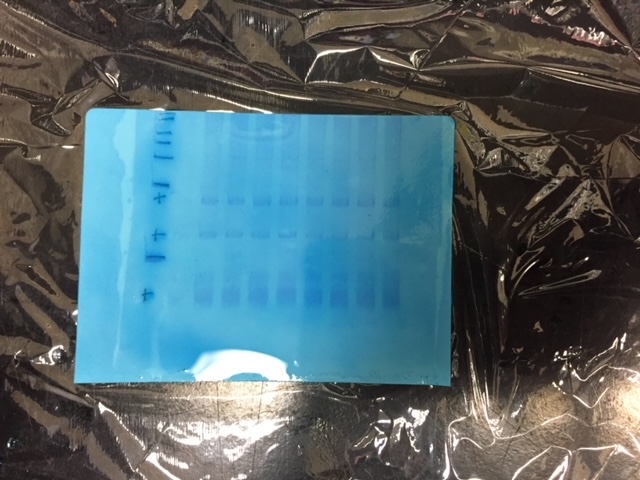 |
| Figure 3E. TMER4 expression from the plasmids in DMEM media transfections. Only the red area was used for the figure. Rotated version of the blot was used for the figure. Blotted for TMER4 probe. | 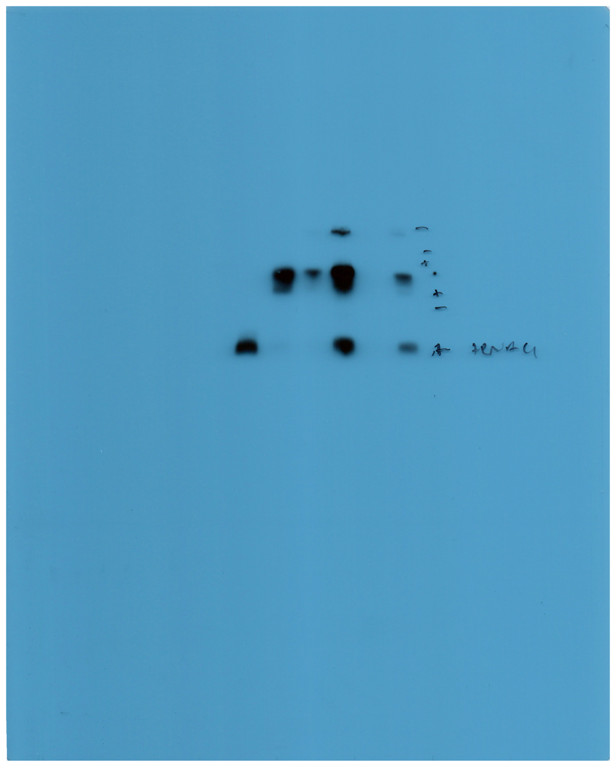 |
| Figure 3E. Loading control. Only the red area was used for the figure. Rotated version of the blot was used for the figure. Blotted for 5S rRNA probe. | 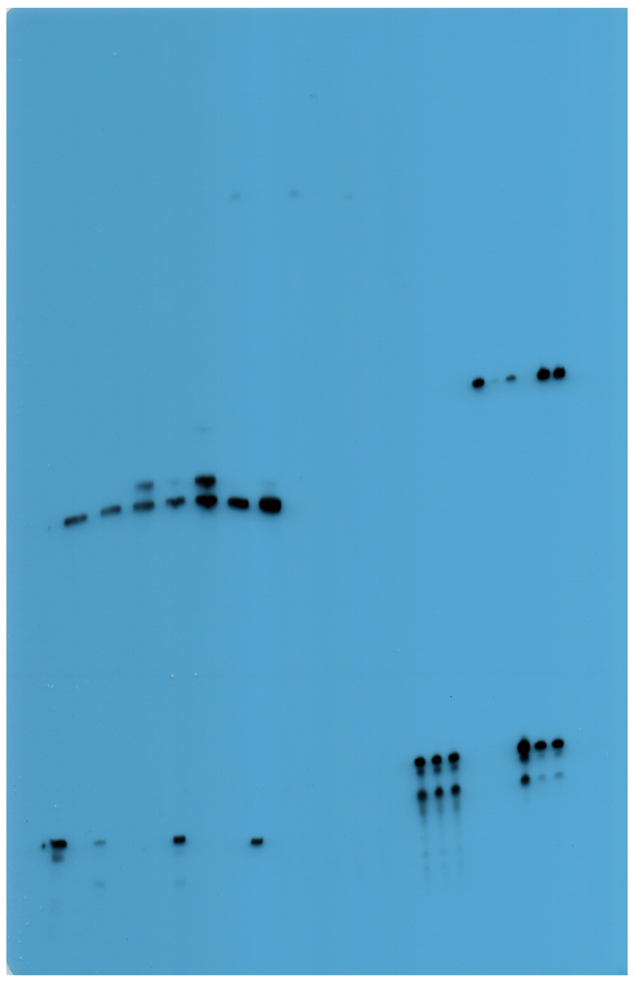 |

# Supplementary Figures and Tables

## Supplementary Figures


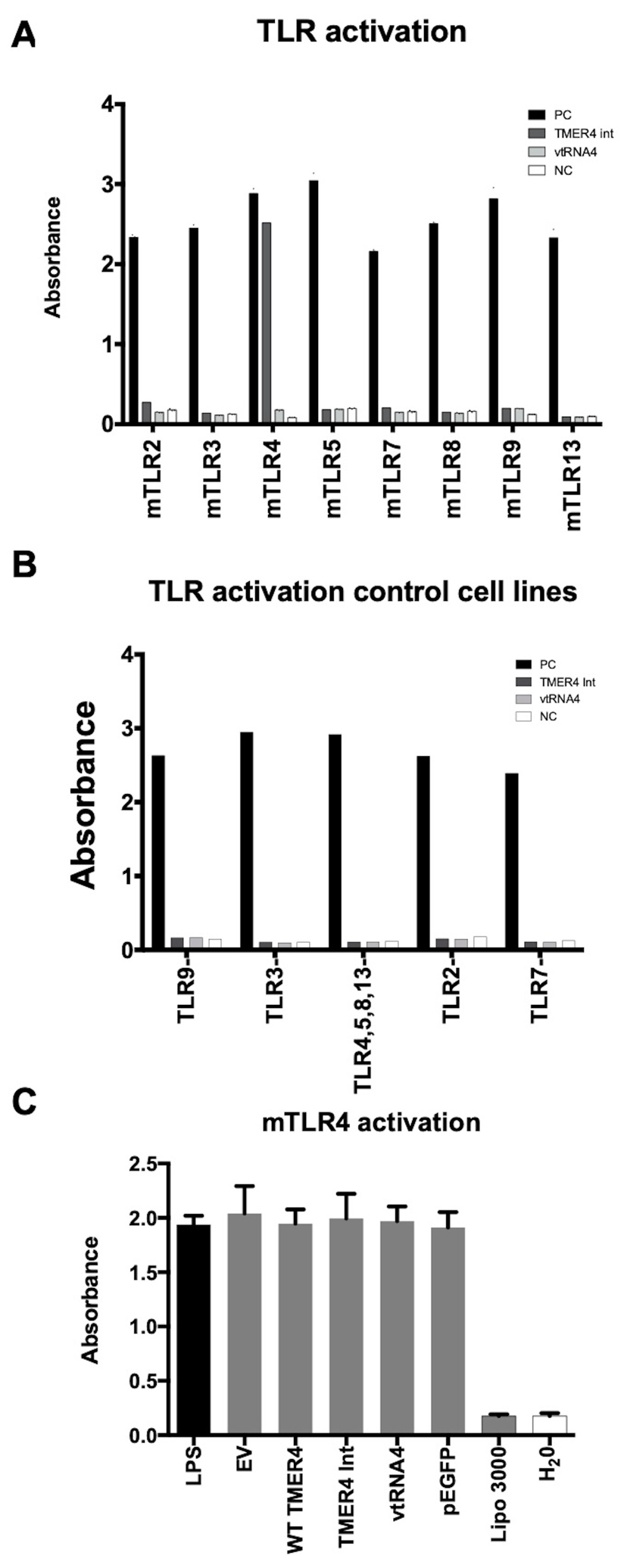


**Supplementary Figure 1.** TLR screening performed at Invivogen and standard plasmid preparations induce TLR4 signaling. A. TMER4 intermediate form and viral tRNA4 expressing plasmids were tested by Invivogen mouse TLR panel for activation. Each TLR cell line were transfected with mock control, or the corresponding plasmid. The positive controls for each cell line are given in the Supplementary Table. B. The control cell lines do not express the TLR therefore induced with TNF alpha at 100ng/mL. C. NucleoSpin preparared plasmid preparations were tested in our laboratory for TLR4 induction.

**Supplementary Figure 2.** TLR4 induction assay with EBV EBER1/2 and Adenovirus VA RNA plasmids. Corresponding plasmids were transfected into HEK-Blue mTLR4 cell lines and absorbance was measured 24 hours after transfection.


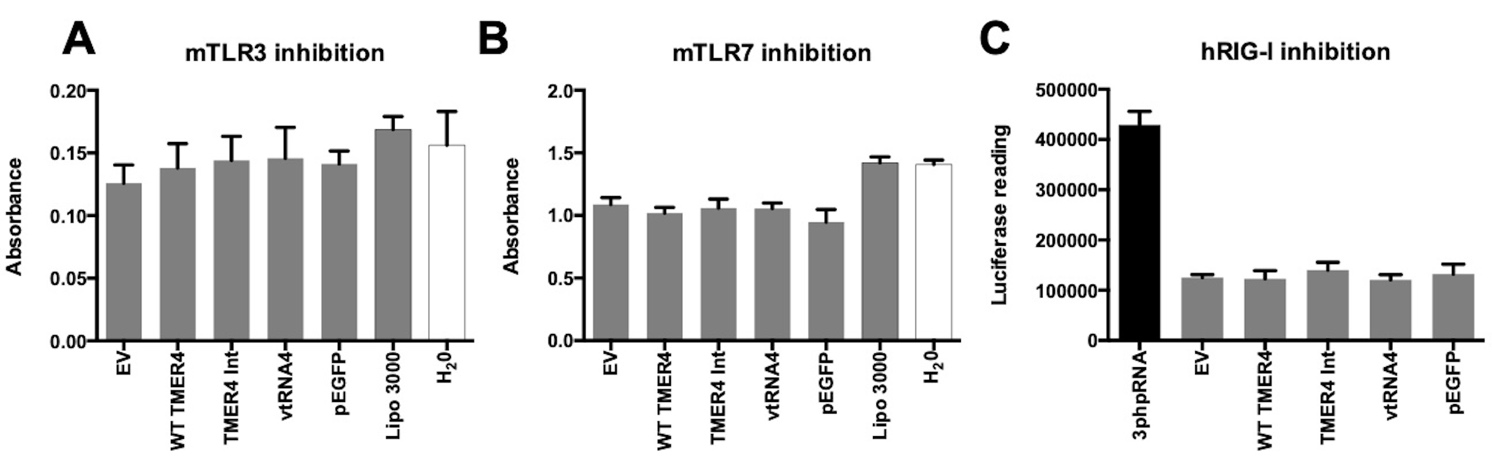
 Supplementary Figure 3. mTLR3, mTLR7 and hRIG-I inhibition by TMER noncoding RNA.A. mouse TLR3 induction with transfection of plasmids had no inhibitory effect on the induction of TLR3 pathway. 100ng/mL polyI:C is used to induce transfected cells. B. mouse TLR7 is not inhibited viral noncoding RNAs. 100ng/mL CL307 reagent is used to induce transfected cells. C. Transfection of plasmids had inhibited activation of RIG-I pathway even with the control plasmids. The small reduction of plasmid transfected cells ability to respond to stimuli is most likely results from the stress of transfection of plasmids themselves because no difference was observed between the empty vector versus the noncoding RNA encoding plasmids. 10ng of 3p-hpRNA is used for each well.

**
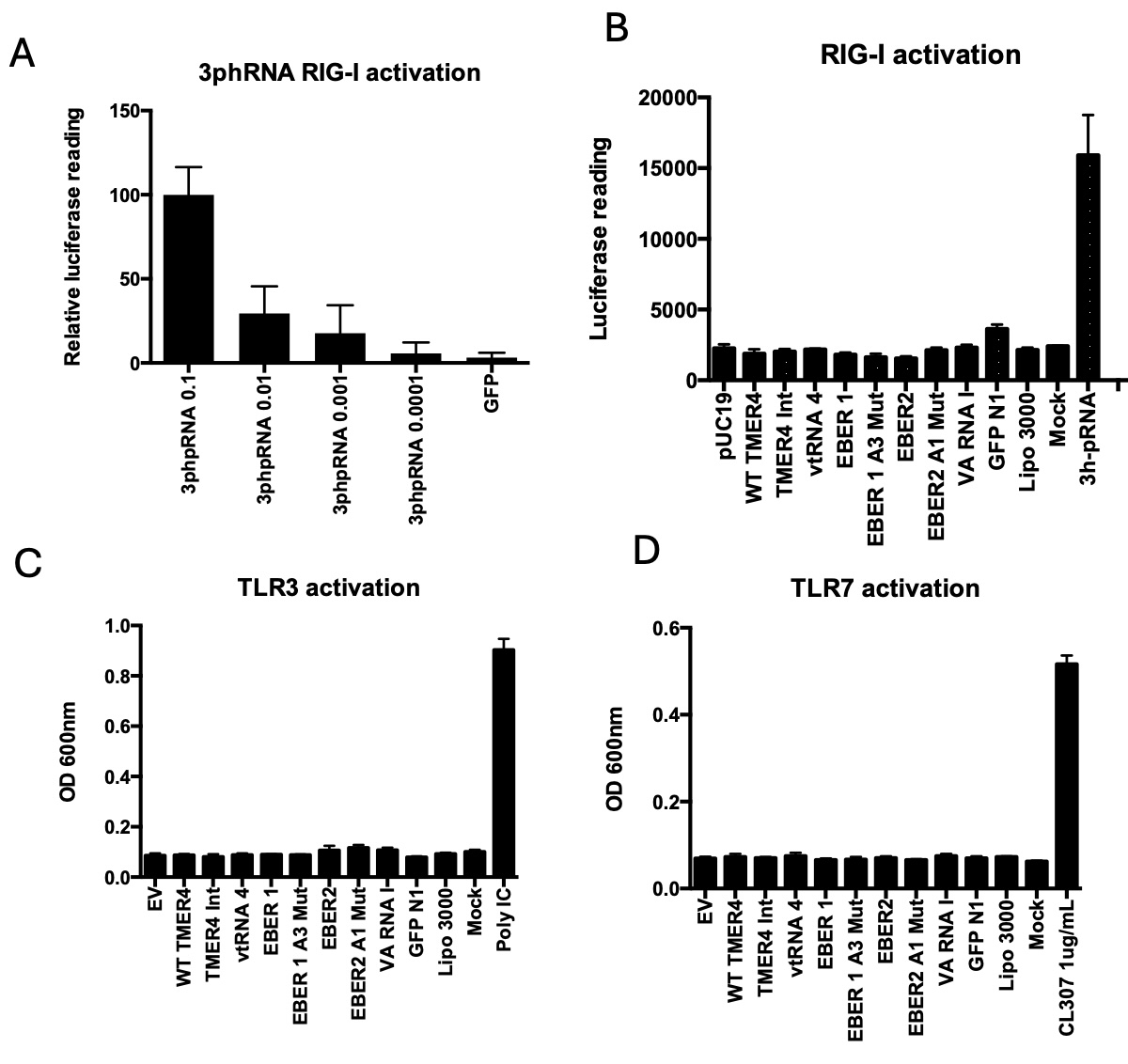
Supplementary Figure 4.** TLR3,7 and RIG-I cell lines activation assay with EBV EBER1/2 and Adenovirus VA RNA 1. A. Decreasing amounts of 3phRNA (0.1 ug to 0.1 ng) was transfected into HEK RIG-I Lucia cell lines and luciferase reading were observed. B. Plasmids encoding EBER1/2 and their mutated versions, and VA RNA 1 were transfected into HEK RIG-I Lucia cells lines and luciferase readings were recorded. 10 ng of 3phpRNA was used as a control. C-D. Plasmids encoding EBER1/2 and their mutated versions, and VA RNA 1 were transfected into HEK Blue TLR3 and 7 cell lines and 24 hours after transfection absorbance was measured. None of the tested plasmids induced the reporter cell lines. As controls, 100 ng/mL Poly IC was used to induce TLR3 and 1 ug/mL CL307 agent was used to induce TLR7 cell lines.

## Supplementary Tables

### Supplementary Table 1

ncRNA sequences used in this study are provided.

| ncRNA | Sequence |
| --- | --- |
| vtRNA4 | GTCGGGGTAGCTCAATTGGTAGAGCGGCAGGCTCATCCCCTGCAGGTTCTCGGTTCAATCCCGGGTCCCGACGC |
| TMER4 intermediate | GTCGGGGTAGCTCAATTGGTAGAGCGGCAGGCTCATCCCCTGCAGGTTCTCGGTTCAATCCCGGGTCCCGACGC*****AGAGTTGAGATCGGGTCGTCTCCCCCTGGCGGAAGGAGGCAAACCCGAGCTCCTCC |
| TMER4 full length | GTCGGGGTAGCTCAATTGGTAGAGCGGCAGGCTCATCCCCTGCAGGTTCTCGGTTCAATCCCGGGTCCCGACGCAGAGTTGAGATCGGGTCGTCTCCCCCTGGCGGAAGGAGGCAAACCCGAGCTCCTCCTTCTTTTCAACCACCTCCCACAATTTCAGAGTCTTAGCCAGATTATCTGAAACTGTGTGAGGTGGTTTTTTCT |
| EBER1 | AGGACCTA**CGCTGCCCTAGAGGTT**TTGCTAGGGAGGAGACGTGTGTGGCTGTAGCCACCCGTCCC**GGGTACAAGTCCC**GGGTGGTGAGGACGGTGTCTGTGGTTGTCTTCCCAGACTCTGCTTTCTGCCGTCTTCGGTCAAGTACCAGCTGGTGGTCCGCATGTTTT |
| EBER2 | AGGACAGCC**GTTGCCCTAGTGGTT**TCGGACACACCGCCAACGCTCAGTGCGGTGCTACCGACCCG**AGGTCAAGTCCC**GGGGGAGGAGAAGAGAGGCTTCCCGCCTAGAGCATTTGCAAGTCAGGATTCTCTAATCCCTCTGGGAGAAGGGTATTCGGCTTGTCCGCTATTTTT |
| VA RNA I | AGCGGGCACTCTTCCGTGGTCTGGTGGATAAATTCGCAAGGGTATCATGGCGGACGACCGGGGTTCGAGCCCCGTATCCGGCCGTCCGCCGTGATCCATGCGGTTACCGCCCGCGTGTCGAACCCAGGTGTGCGACGTCAGACAACGGGGGAGTGCTCCTTTT |

The star in the TMER4 intermediate sequence displays where the CCA sequence was inserted. In EBER sequences bold underlined sequences are mutated to destroy polymerase III promoter sites.

### Supplementary Table 2

## Ligands and corresponding concentrations used to induce TLRs.

| Receptor | Ligand | Concentration |
| --- | --- | --- |
| mTLR2 | HKLM (heat-killed *Listeria monocytogenes*) | 1x108 cells/mL |
| mTLR3 | Poly(I:C) HMW | 1 μg/mL |
| mTLR4 | *E. coli* K12 LPS | 100 ng/mL |
| mTLR5 | *S. typhimurium* flagellin | 100 ng/mL |
| mTLR7 | CL307 | 1 μg/mL |
| mTLR8 | CL075 + Poly(dT) | 10 μg/mL, 10 nM respectively |
| mTLR9 | CpG ODN 1826 | 1 μg/mL |
| mTLR13 | ORNSa19 | 200 ng/mL |

8 different mouse Toll like receptors and their ligands at given concentrations used by the Invivogen company for the initial screening studies.
